# New Prognostic Biomarkers in Metastatic Castration-Resistant Prostate Cancer

**DOI:** 10.3390/cells10010193

**Published:** 2021-01-19

**Authors:** Vincenza Conteduca, Alessandra Mosca, Nicole Brighi, Ugo de Giorgi, Pasquale Rescigno

**Affiliations:** 1 Department of Medical Oncology, Istituto Scientifico Romagnolo per lo Studio e la Cura dei Tumori (IRST) IRCCS, 47014 Meldola, Italy; vincenza.conteduca@irst.emr.it (V.C.); nicole.brighi@irst.emr.it (N.B.); ugo.degiorgi@irst.emr.it (U.d.G.); 2 Multidisciplinary Outpatient Oncology Clinic, Candiolo Cancer Institute, FPO-IRCCS, Candiolo, 10060 Turin, Italy; alessandra.mosca@ircc.it; 3 Interdisciplinary Group for Translational Research and Clinical Trials, Urological Cancers (GIRT-Uro), Candiolo Cancer Institute, FPO-IRCCS, Candiolo, 10060 Turin, Italy

**Keywords:** prostate cancer, androgen receptor, PTEN, DNA repair defects, prognostic biomarkers

## Abstract

Prostate cancer is one of the most frequent cancers in men and is a common cause of cancer-related death. Despite significant progress in the diagnosis and treatment of this tumor, patients who relapse after radical treatments inevitably develop metastatic disease. Patient stratification is therefore key in this type of cancer, and there is an urgent need for prognostic biomarkers that can define patients’ risk of cancer-related death. In the last 10 years, multiple prognostic factors have been identified and studied. Here, we review the literature available and discuss the most common aberrant genomic pathways in metastatic castration-resistant prostate cancer shown to have a prognostic relevance in this setting.

## 1. Introduction

Prostate cancer (PC) is the second most common cancer for incidence and the fifth most frequent cause of cancer-related mortality in men worldwide, while in Western countries it represents the second cause of death for cancer in males [1,2]. The incidence of PC has been rising in recent years, due to the broader availability of PSA screening in Western countries’ populations [3].

Prostate cancer is a highly heterogeneous disease, in both clinical and molecular aspects. PC may range from slowly progressing to very aggressive disease, mostly due to the development of resistance to treatment. Although advanced PC typically starts with a strong dependency on androgens, resulting in initial responses to androgen deprivation treatments, most patients progress to castration-resistant disease (CRPC) [4]. CRPC status is defined as the presence of castrate serum testosterone levels (<50 ng/dL or 1.7 nmol/L) plus either biochemical progression (three consecutive rises in prostate-specific antigen (PSA) 1 week apart, resulting in two 50% increases over the nadir, and PSA >2 ng/mL) or radiologic progression (consisting of the appearance of new lesions, such as two or more new bone lesions on bone scan or a soft tissue lesion using the Response Evaluation Criteria in Solid Tumors) [5].

In addition to the different possible clinical presentations, intra-patient tumor heterogeneity and clonal evolution must be taken into account and represent a challenge in the management of patients. Several recurrent molecular pathways have been identified in the metastatic CRPC (mCRPC) molecular landscape, resulting in resistance to treatments and tumor progression, and impacting patients’ survival. These include androgen receptor (AR) aberrations, PTEN loss, DNA repair gene deletions, *TP53* mutations, and *RB1* loss [6,7].

## 2. Clinical Studies of Circulating Androgen Receptor (*AR)* Status in Liquid Biopsy as a Prognostic Biomarker in mCRPC

Androgens are involved in the normal development of the prostate gland, but also in prostate carcinogenesis. In normal prostate tissue, differentiation is promoted, while during the progression of PC, proliferation gradually overcomes differentiation. AR contributes to the control of the balance between cell proliferation and differentiation [8,9,10,11].

**Figure 1:**
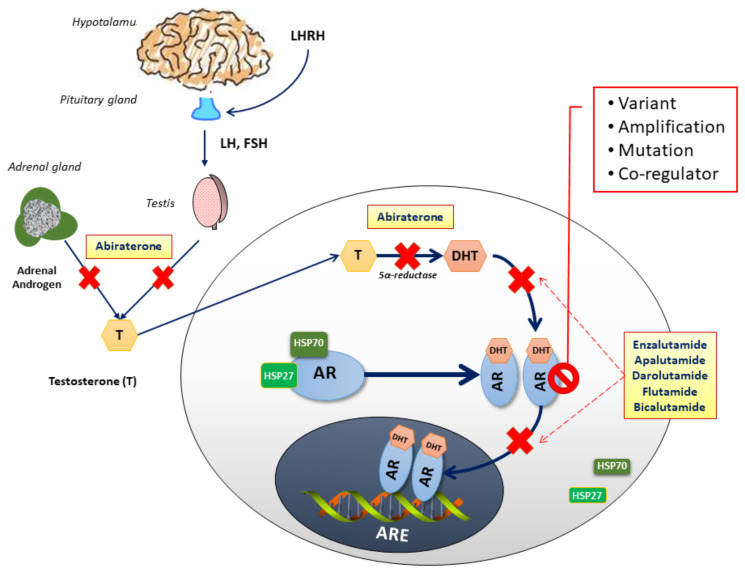
Androgen-dependent signaling through the androgen receptor (AR). After its production in the testes ad the adrenal glands, testosterone (T) is converted by 5α-reductase to dihydrotestosterone (DHT), its active metabolite. Generally, androgens bind to AR, dissociating chaperone proteins, including members of the heat shock protein family (HSP27 and HSP70). Upon homodimerization, ligand-bound AR dimers translocate to the nucleus where they bind to androgen response elements (ARE), acting as transcription factors to downstream targets. AR-directed drugs are illustrated at their points of pathway alteration: Abiraterone disrupts the androgen biosynthesis inhibiting the 17α-hydroxylase/C17,20-lyase (CYP17), an enzime expressed in testicular, prostate, and adrenal tissue; Flutamide, and Bicalutamide, reversibly, and Enzalutamide, Apalutamide, Darolutamide, irreversibly, prevent testosterone binding to the AR, incapacitating its translocation to the nucleus.

Androgen signaling inhibition, either through androgen deprivation or AR activity block, is therefore the mainstay of PC treatment. In the last decade, novel hormonal drugs have been approved in castration-sensitive prostate cancer (CSPC) and/or mCRPC thanks to their mechanisms of action (abiraterone prevents androgen biosynthesis, and enzalutamide, apalutamide and darolutamide inhibit AR translocation to the nucleus) (Figure 1).

Indeed, even when the disease turns castration-resistant, AR remains an important molecular driver [12,13,14,15]. Subsequently, the disease acquires resistance to both androgen deprivation therapies (ADT) and AR-directed treatments through molecular pathways mostly driven by AR aberrations. These may include *AR* gene mutations, AR splice-variants, an *AR* gene amplification; also, the presence of AR co-regulators may contribute to resistance to treatments [16,17]. These aberrations are seen to increase during tumor progression: in the setting of castration resistance, they are reported in 10–15% of patients, while they are identified in up to 40% of cases in patients treated with second or further treatment lines for advanced CRPC [18].

The vast majority of patients with AR pathway alterations present *AR* gene amplifications (or gain) [6]. The efficacy of antagonists in inhibiting a receptor-ligand interaction depends greatly on the concentrations of receptors, agonists, and antagonists. Thus, in the presence of *AR* gene amplifications, AR antagonists (such as enzalutamide, apalutamide and darolutamide) may be unable to effectively antagonize AR proteins. Moreover, since PC is able to synthetize androgens, even when androgen concentrations are under castrate levels, AR signaling still remains active. Through these mechanisms, *AR* amplifications and enhanced AR signaling result in resistance to first- and second-generation antiandrogens [19,20].

Other AR alterations such as *AR* point mutations [16] can also result in treatment resistance. Several point mutations have been described: the most clinically relevant are T878A (formerly F876L and T877A) and W742C, affecting the ligand binding domain and conferring resistance to flutamide and bicalutamide; the point mutation F877L, instead, results in resistance to apalutamide and enzalutamide. Mutations of T878A or L702H have been reported to have a role in abiraterone resistance where *AR* T878A enables the activation of the receptor by progesterone and L702H the activation by glucocorticoids such as prednisone, which is given together with abiraterone and docetaxel. Other mutations frequently described include V715M, V730M, and H875Y [7,8,21].

Secondary AR alterations, usually emerging after treatment with abiraterone or enzalutamide, are represented by AR splice variants (AR-Vs). AR-V7 is the most frequent variant, conferring a constitutive activation resulting in enhanced transcription, cell proliferation and DNA repair. Thus, AR-Vs are related to treatment resistance and poor outcomes [22,23,24]. However, AR-Vs’ activity is usually seen in the presence of *AR* amplifications [4,25].

Finally, the presence of AR co-regulators represents another possible mechanism of resistance to treatments. Several co-activators may interact with AR and enhance transcription. Among them, the activity of the members of the p160 steroid receptor coactivator (SRC) family, the histone acetyltransferase CBP/p300, and the pioneer factor forkhead box A1 (FOXA1) have been described to be associated with treatment resistance and worse prognosis [17,26,27].

All these alterations involving the AR pathway can be studied using liquid biopsies. For example, given the heterogeneity of AR-V7 expression at different metastatic sites [28], its study in circulating tumor cells (CTCs) could provide a comprehensive assessment of the AR-V7 status and a monitoring of AR-V7 changes during treatment. The first study by Antonarakis et al. [24] showed that AR-V7 positive CTCs were associated with resistance to second-line AR-directed therapies in mCRPC patients. Of the 62 enrolled patients, 19% of the 31 abiraterone-treated patients and 39% of the 31 enzalutamide-treated patients had AR-V7-positive CTCs, respectively. The AR-V7-positive patients presented significantly worse outcomes, including lower PSA response rates and worse progression-free survival (PFS) and overall survival (OS). In a follow-up study on 202 prospectively enrolled patients, treated with either abiraterone or enzalutamide, AR-V7 positivity was significantly associated with other adverse prognostic factors, including a Gleason score of ≥8, the presence of metastatic disease, and previous treatment with AR-directed therapies and taxanes. On multivariable analysis, the prognostic impact of AR-V7-positivity on PFS and OS was confirmed [29]. The same group also investigated AR-V7 mRNA expression as a predictor of outcomes in mCRPC patients treated with taxanes [30]. The study enrolled 37 patients, of which 46% had AR-V7-positive CTCs. Their outcomes were, however, not dissimilar to those patients with AR-V7-negative CTCs, suggesting that AR-V7 status was not predictive of taxane resistance. On the contrary, the AR-V7-positive patients treated with taxanes had significantly better outcomes than the AR-V7-positive men who had received AR-targeting agents, suggesting that taxane therapy is preferable for AR-V7-positive mCRPC patients. Similar results were observed in a study of 29 mCRPC patients receiving cabazitaxel [31]. Recently, Scher et al. also reported an automated method of detecting intranuclear AR-V7 protein using immunofluorescent staining of CTCs that was also predictive of the clinical response to second generation hormonal treatments, as shown in the tissue-based study of AR-V7 expression [32]. Moreover, nuclear localization of the AR-V7 protein was an independent prognostic factor for survival (HR 2.38; 95% CI, 1.02–5.53; *p* = 0.045) in taxane-treated patients. Using the same automated immunofluorescent assay of CTC, a multicenter study also confirmed the predictive value of AR-V7 positivity in 142 mCRPC patients, showing a benefit in OS for AR-positive patients treated with chemotherapy compared to those treated with AR-directed agents [33].

Interestingly, in the TAXNERGY study (early switch from first-line docetaxel/prednisone to cabazitaxel/prednisone and the opposite sequence, exploring molecular markers in mCRPC patients), using a novel digital droplet PCR assay, 67% of the 54 docetaxel/ or cabazitaxel treated patients had AR-V7-positive CTCs [34]. These patients presented with lower PSA response rates (78% vs. 58%; *p* = 0.23) and shorter median PFS (8 vs. 12 months; HR, 0.38; *p* = 0.01) compared to the AR-V7-negative patients.

Preliminary findings were also reported in 15 AR-V7-positive patients treated with ipilimumab and nivolumab showing lack of efficacy in this subset of patients, with only two patients (13%) achieving a PSA response [35].

Further circulating biomarkers in CRPC are represented by *AR* gene aberrations that are infrequent in the early stages, but common in the CRPC setting. Therefore, NGS- and PCR-based studies have been particularly focused on *AR* copy number in advanced disease. Romanel et al. [36] analyzed 274 plasma samples from 97 CRPC patients treated with abiraterone. The authors reported plasma *AR* gain and point mutations to be mutually exclusive. While *AR* copy number was not different during treatment, the emergence of T878A or L702H AR amino acid changes was reported in 13% of samples upon progression. There was also an association between plasma AR aberrations and worse OS (HR 7.33, 95% CI 3.51–15.34, *p* = 1.3 × 10^−9^) and PFS (HR 3.73, 95%CI 2.17–6.41, *p* = 5.6 × 10^−7^). Subsequently, a multicenter biomarker study assessed the role of plasma AR also in chemotherapy-naïve patients [37]. There was a primary cohort of 73 chemotherapy-naïve and 98 post-docetaxel patients treated with enzalutamide or abiraterone and a secondary cohort of 94 chemotherapy-naïve patients treated with enzalutamide in 16 institutions in the PREMIERE trial (NCT02288936). In the primary cohort, 14% pre-chemotherapy and 34% post-docetaxel patients presented *AR* gain, while 11% post-docetaxel but no chemotherapy-naïve abiraterone-treated patients showed *AR* point mutations. Chemotherapy-naïve and post-docetaxel AR-gained patients demonstrated a poor survival (OS: HR = 3.98, 95%CI 1.74–9.10, *p* < 0.001 and HR = 3.81, 95%CI 2.28–6.37, *p* < 0.001, respectively, and PFS: HR = 2.18, 95%CI 1.08–4.39, *p* = 0.03, and HR = 1.95, 95%CI 1.23–3.11, *p* = 0.01, respectively). Patients with *AR* mutations also showed a significantly worse OS (HR = 3.26, 95%CI 1.47–not reached, *p* = 0.004). In the validation cohort, 11 (12%) patients had *AR* gain. AR-gained patients had a reduced biochemical PFS (HR = 4.33, 95%CI 1.94–9.68, *p* < 0.001), radiographic PFS (HR = 8.06, 95%CI 3.26–19.93, *p* < 0.001), OS (HR = 11.08, 95%CI 2.16–56.95, *p* = 0.004).

As previous findings have suggested that the detection of AR-V7 in CTCs could predict a response to AR-signaling inhibitors versus taxane chemotherapy, recently, the role of cell-free *AR* copy number has also been investigated in CRPC patients treated with taxanes [38,39]. A biomarker study of 163 docetaxel-treated patients assessed the association between plasma *AR* and outcomes in mCRPC, reporting only a significant worse OS in AR-gained patients (HR = 1.61, 95%CI 1.08–2.39, *p* = 0.018). Moreover, the same authors incorporated updated data from their prior study [38], and interrogated the interaction between plasma *AR* and treatment type in the abiraterone/enzalutamide-treated group (73 patients), after and in 115 first-line docetaxel patients. The findings from this study would suggest that AR-normal men could benefit from hormonal treatment in the first-line therapy group, while plasma AR-gained patients had a better response to docetaxel. Similarly, in a subsequent work with mCRPC patients receiving second-line cabazitaxel therapy, a significant treatment interaction between plasma *AR* and cabazitaxel vs. AR-directed therapies was observed for OS (*p* = 0.041). An exploratory analysis showed AR-gained patients treated with an initially reduced dose of cabazitaxel presented a meaningfully worse OS/PFS and would probably need a standard initial dose of cabazitaxel. This study suggests that plasma *AR* may improve clinical decision-making in choosing between adapted and standard regimen of taxanes [39].

Recently, plasma *AR* gene status has been also explored in a phase 2 clinical trial (NCT03454750) of mCRPC patients treated with ^177^Lu–PSMA–617 [40]. Early progressive disease was observed in 17 (42.5%) of the 40 patients (12 of 15 (80%) with *AR* amplification and 5 of 25 (20%) with *AR* normal (*p* = 0.0002)). The OR for patients with early disease progression and AR gain was 16.00, 95% CI 3.23–79.27, *p* = 0.0007. These preliminary data suggest that plasma *AR* status assessed by ddPCR could be helpful to identify resistance to ^177^Lu–PSMA–617 in mCRPC patients.

Recent studies determined the impact of *AR* mutations, e.g., F877L and T878A, responses to novel AR-directed therapies, such as apalutamide [41] and darolutamide [42]. The phase I/II study ARN-509-001 [40] used the sensitive BEAMing assay to detect AR mutations in cell-free DNA in apalutamide-treated nonmetastatic CRPC and mCRPC patients. However, the overall frequency of *AR* mutations was so low that no conclusions could be drawn.

A recent paper [43] uncovered an orthogonal methylation signature in 25 mCRPC patients treated with abiraterone or enzalutamide. The study showed an association between *AR* copy number gain and an enrichment for AR binding sequences and hypomethylation of these segments. Moreover, plasma methylome analysis could accurately estimate tumor fraction. All these findings permitted to identify patients with a more aggressive clinical course based on this methylation pattern and so a molecular prognostic stratification of mCRPC individuals.

The role of plasma DNA analysis has been recently investigated in combination with functional imaging and other routinely obtained circulating biomarkers in order to improve prognostication of mCRPC in patients [44]. This work provided innovative insights on utility of integrating functional imaging with plasma DNA analysis including AR status assessment and other noninvasive biomarkers to improve treatment.

## 3. The Prognostic Role of PTEN Alterations and Its Pathway

Alterations in phosphatase and tensin homolog (PTEN) and activation of the kinases PI3K and AKT, downstream of its axis, are common in primary PC and are enriched in mCRPCs [6,45]. Genomic inactivation of *PTEN*, mainly through the loss of its locus on 10q23.31, is the most common molecular aberration involving this pathway occurring in approximately 40% of CRPCs [6,7,45]. However, mutations and complex genomic rearrangements can also occur [46], as well as aberrations in *PIK3CA, PIK3CB, PIK3R1*, *PIK3R3* and *AKT1* [6]. All these alterations often result in the activation of this pathway and are responsible for cell growth, cell cycle progression, and cell proliferation [47]. The PTEN/PI3K/AKT pathway indirectly also regulates the AR signaling through a negative feedback loop [48,49], and this is particularly relevant for the therapeutic approaches in mCRPC. Immunohistochemistry (IHC) studies have consistently shown PTEN loss as a poor prognostic factor in mCRPC [50,51,52,53].

Ferraldeschi et al. analyzed the PTEN status of 144 patients who had received abiraterone post-docetaxel. In this retrospective study, the loss of PTEN expression was not only associated with shorter median OS (14 vs. 21 mo; hazard ratio [HR]: 1.75; 95% confidence interval [CI], 1.19–2.55; *p* = 0.004), but also with shorter median duration on abiraterone (24 vs. 28 wk; HR: 1.6; 95% CI, 1.12–2.28; *p* = 0.009). More importantly, PTEN status was consistent between matched CSPC and CRPC tumor biopsies in nearly 90% of cases [52], meaning that the loss of PTEN is an early event in prostate cancer tumorigenesis. Similarly, *PI3K* and *AKT* alterations were identified in 11% of 418 prostate cancer tissue samples, of these, 26/418 (6%) were pathogenic pathway activating mutations. These mutations were associated with a shorter OS (2.8 vs. 4.3 years; HR: 2.73; *p* < 0.001) and duration of abiraterone/enzalutamide (5.9 vs. 10.0 mo; *p* < 0.001) [54].

Altogether, these data highlight the relevance of the aberrations in the PTEN pathway as prognostic factor in mCRPC. However, more recently, these alterations have been studied as biomarkers of response to the combination of new generation hormonal treatments and AKT inhibitors such as ipatasertib and capivasertib [55,56].

## 4. DNA Repair Defects as Prognostic Biomarkers in mCRPC

Genomic instability is a common denominator of many different cancers. This instability derives from the high rate of cell division that is responsible for the fast accumulation of genomic aberrations [57]. Therefore, defects in the DNA damage repair (DDR) play a key role in the promotion of cancer growth [58]. Most of the endogenous or exogenous mutagens can cause DNA single-strand breaks (SSBs), although double-strand breaks (DSBs) are more lethal to cells. Thus, most DDR-directed therapies target the repair mechanisms associated with DSBs, increasing replication stress; or inhibit cell cycle checkpoints that facilitate DNA repair. More specifically, defects in (or inhibition of) the high-fidelity DDR system, such as homologous recombination (HR), increase genomic instability, since cells will try to rely on compensatory mechanisms of repair that are often error-prone in order to survive [59].

Breast cancer-associated gene2 (*BRCA2*), a key member of the HR and Fanconi anemia complex, is the most commonly mutated DDR gene in prostate cancer. In the metastatic setting, germline *BRCA2* (*gBRCA*) mutations have a prevalence of 3–6%, while somatic mutations and homozygous deletions account for ~20% of metastatic CRPC (mCRPC) cases [6,7,19,60]. On the other hand, alterations in other HR members, *BRCA1*, *PALB2* and *RAD51*, are present in <1% of mCRPC. The prognostic role of germline and somatic mutations in HR genes, and specifically of *BRCA2* alterations, has been investigated in detail in recent years. *BRCA2* mutation carriers have a 5-year prostate cancer-specific survival (CSS) rate of ~50%, progressing rapidly from localized PC to mCRPC [61,62,63,64]. Similarly, in the PROREPAIR-B study, which prospectively followed a cohort of 419 gDDR-defective mCRPC patients in order to evaluate the impact of these alterations on outcomes, *gBRCA2* patients were found to have a shorter CSS compared to non-carriers (17.4 months vs. 33.2 months, *p* = 0.027) [65]. A recent meta-analysis of 10 studies including 525 *BRCA2* mutation-carriers and 8,463 non-carriers confirmed that carrying a *BRCA2* alteration correlates with a reduced CSS and OS (Hazard Ratios (HRs) 2.53 vs. 2.21 respectively, *p* < 0.001). The results also demonstrated that *BRCA2*-mutation carriers were characterized by a higher Gleason Score (GS) (>7), TNM stage (>T3, N1, M1) at diagnosis [66]. The predictive role of these mutations as biomarkers of response to standard treatments for mCRPC remains, however, controversial [67,68,69]; nevertheless, it is undoubted that BRCA2 alterations predict response to PARP inhibitors in mCRPC [70,71].

*ATM* (ataxia telangiectasia, mutated) is a member of the PI3 family of serine-threonine kinases and functions as a DNA damage sensor [72]. Therefore, it is involved in DNA repair, triggering the action of others DDR proteins [73]. Its alterations are found in nearly 10% of mCRPC patients [6,60], but its loss does not determine the same mutational signature as *BRCA1* or *BRCA2* biallelic loss [74,75]. Its prognostic value is unclear, but ATM loss does not seem to affect mCRPC patients’ outcome [76].

CDK12 is reportedly involved in regulating expression of several HR genes [77]; somatic defects in *CDK12* are associated with a genome-wide focal tandem duplication (FTD) signature [78]. Multiple retrospective studies have reported a worse survival for *CK12* mutant mCRPC, which are also characterized by a high Gleason score (>8) and a tumor immune infiltrate, potentially immunosuppressive, comprised of CD4+FOXP3- cells [79]. The FTD phenotype has also been correlated with increased neo-antigen burden in those tumors, positioning *CDK12* among the putative biomarkers of response to novel immunotherapies in mCRPC [74].

The mismatch repair (MMR) system is a post-replicative, high-fidelity, single-strand repair mechanism that recognizes and reverses DNA base mismatches and insertion/deletion loops, compromised MMR results in microsatellite instability, and a hypermutator phenotype that has been associated with chemotherapy resistance but immunotherapy sensitivity [80]. The prevalence of MMR aberrations ranges between 3% and 12%, depending on the assay and population selected [6,81,82], and could be underestimated since alterations in *MSH2* and *MSH6*, the two main players of the MMR system, involve noncoding regions detectable only by whole-genome studies. MMR-defective status (dMMR) was associated with poor OS compared to MMR-proficient tumors (3.8 vs. 7.0 years from start of luteinizing hormone–releasing hormone; *p* = 0.005), increased T-cell infiltration and elevated PD-L1 protein expression in a cohort of 124 mCRPC biopsies from a single institution [83]. Transcriptome analysis has also revealed that cancers with an MMR mutational signature are characterized by increased expression of inferred immune cells, immune checkpoints, and T-cell-associated transcripts [83].

Considering their localization on chromosome 13, *BRCA2* losses often co-occur with the retinoblastoma (*RB1*) ones [84]. *RB1* is a tumor suppressor that also plays a relevant role in mCRPC. Comprehensive genomic, transcriptomic and histologic analysis of 429 patients with mCRPC linked with longitudinal clinical outcomes identified *RB1* genomic alteration as a potent predictor of poor outcome especially when associated with tumor protein p53 (*TP53*) alterations [85]. TP53 plays a role in cell cycle and genomic stability. It is able to activate DNA repair proteins upon DNA damage, arresting cell growth at the G1/S regulation point. In case of irreparable damage, TP53 is able to induce apoptosis and cell death [86] and represent with PTEN one of the most common alteration in mCRPC [6]. The prognostic role of *RB1* and *TP53* aberrations was also confirmed by another large retrospective study in which 470 treatment-naïve prostate cancer diagnostic biopsies and 61 matched mCRPC biopsies were analyzed using targeted and low-pass whole-genome sequencing. Moreover, this study demonstrated that RB1 losses along with TP53 and AR aberrations were enriched in later stages [87].

## 5. Use of Molecular Biomarkers in Localized Prostate Cancer

We explored the relevance of prognostic biomarkers in mCRPC; however, in the last few years, some studies have proposed stratifying patients with localized PC based on molecular biomarkers. Traditionally, Gleason score, clinical staging and PSA at diagnosis have helped classifying localized PCs as being at low, intermediate, and high risk of relapse [88]. Recently several tissue-based multigene expression (Decipher, Oncotype Dx Prostate, Polaris) and a protein-based (ProMark) classifiers have been proposed to identify patients with biologically significant disease [89,90,91,92].

Using RNA analysis on formalin fixed paraffin embedded clinical samples, studying biological pathways with a known role in prostate tumorigenesis (i.e., proliferation, cell cycle, etc.), these classifiers (Decipher, Oncotype Dx Prostate, Polaris) offer scores that, integrating other clinical–pathological features, might help identifying patients with localized disease but with worse outcomes. Potentially, these tools could help clinicians in deciding which patients would benefit from surveillance vs. active treatment, or a salvage approach after a radical treatment [89,90,91,92].

However, tissue-based molecular testing is dependent on tissue preservation and influenced by the heterogeneity of the disease [93]. Moreover, the high costs and the lack of prospective validation in clinical trials represent the main limitations to their use in the daily clinical practice. Therefore, based on these considerations, the American society of clinical oncology (ASCO) has not recommended tissue-based molecular biomarkers for routine use [94].

## 6. Conclusions

**Figure 2:**
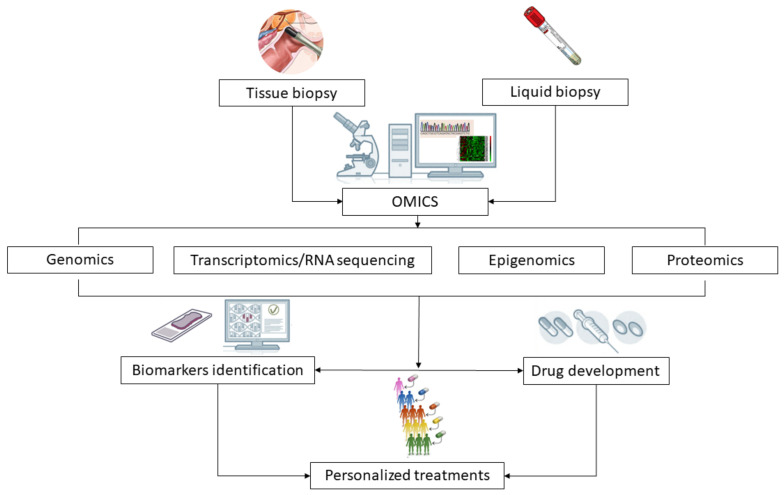
Clinical significance of omics-driven approaches applied to tissue and liquid biopsy analysis in prostate cancer research. The analysis of clinical samples (tissue biopsies, plasma, urine) through the use of novel techniques (such as genomics, transcriptomics, epigenomics and proteomics) allows the identification of prognostic and predictive biomarkers and may guide the development of drugs targeting molecular pathways altered in cancer cells. These advances will contribute to improve patients and treatments selection and to guide personalized interventions for the management of prostate cancer.

Here, we presented the most common genomic pathway aberrations in prostate cancer that have an impact on patient outcomes. All of these studies and data are relevant to understanding the importance of genomic studies in the metastatic setting in order to better evaluate patient prognosis. Since some of these biomarkers can be used as either therapeutic targets or as biomarkers of response to novel agents and standard treatments in mCRPC, they also have emerging potential in targeted therapy and therapy response prediction (Figure 2).
